# Topics of Public Interest

**Published:** 1913-11

**Authors:** 


### TOPICS OF PUBLIC INTEREST

THE DIVINING ROD. One of the silliest superstitions of the ignorant, was that certain persons, armed with a willow or hazel stick, could walk over the surface of the ground and locate, by the turning of the rod, a subterranean supply of water. A congress of scientists recently met at Halle, Germany, to consider the claims of the divining rod, and it has endorsed the superstition as a scientific and practical fact. Not only water, but two beds of potash and one of rock salt were located by the divining rod.

TRANSMUTATION OF ELEMENTS. In connection with the above, attention may again be called to the degradation of elements apparently demonstrated in studies of radio-active substances. Such results of modern science, not to mention the wonderful achievements along purely physical lines resulting in inventions that have revolutionized our ideas of what is and what is not possible, render it hard to draw the line between a proper degree of skepticism and credulity.

THE MANN ACT. The Diggs and Caminetti cases resulted in convictions, in spite of testimony that showed that there was nothing approaching white slavery in the ordinary sense. A Kansas judge has discharged a prisoner on the ground that the law was intended to cover cases in which women were exploited for business purposes and not cases of immorality. A Philadelphia judge has upheld the broad interpretation of the original cases. We were rather surprised to hear an orthodox clergyman condemn the convictions. He held that the law should not deal with cases of immorality as such and that the rulings favored black mail. We can see no sense in distinguishing between interstate immorality and intrastate or intramural immorality. And, in a good many cases there is hypocrisy in the theory that the man is the offender and the woman the innocent victim. We doubt very much whether many persons will be made moral, temperate or otherwise good, by passing laws to correspond to ethical conceptions.

A POLICEWOMAN has been appointed in Rochester.

A Collection of Radiograms, for lantern and sterescopic use, has been begun by the Army Medical Museum at Washington, with the co-operation of various X-ray workers. We would like to see Western New York represented in this movement, not only because our territory contains several excellent radiographers, but because it offered one of the first martyrs to this phase of science, Dr. Louis Weigel of Rochester and Buffalo. And we have a personal interest in the matter, having first demonstrated in this country that the stomach could be mapped out by the fluoroscope without the introduction of instruments.

---

Guinea Pigs. Those interested in raising guinea pigs should write to the U. S. Department of Agriculture at Washington for Bulletin 525, prepared by David E. Lantz. Physicians are frequently asked to suggest employment for semi-invalids, widows left without means of support, etc. The raising of guinea pigs may be available in such cases.

---

Around the World in Eighty Days was the title of one of Jules Verne's novels, popular in the eighties, and still much read. We believe it was the only one of his novels that did not deal with what was considered at the time, an absolute impossibility, though his Twenty Thousand Leagues Under the Sea may now be regarded as merely a forecast of the physically possible. At any rate, the former book was considered a wild flight of the imagination, and even the author made his hero win his race against time by the narrow margin of the day lost on account of going against the apparent revolution of the sun about the earth. Recently, the earth has been toured in 35 days, 21 hours and 35 minutes, at approximately the latitude followed by Verne's hero.

---

White Phenolphthalein. We have recently been supplied with tablets of white phenophthalein. It was not surprising that phenolphthalein could be rendered colorless by the addition of a small amount of some acid substance, but these tablets did not turn pink in an excess of alkali. The manufacturer insists that the tablets do contain phenolphthalein. A chemist, consulted about the matter declares that phenolphthalein does not form any combination of the nature of a salt and that the characteristic indicator reaction would be given with an alkali. We submit the question for discussion.

---

Clinical Lectures on Skin Diseases. The N. Y. Skin and Cancer Hospital announces the fifteenth annual course by Dr. L. Duncan Bulkley, on Wednesday afternoons at 4:15, in the out-patient hall of the hospital. The series begins November 5, and is free to the profession on presentation of their office cards.

---

A Case Illustrating Great Tenacity of Life. No, this is not the one to which most of our exchanges have alluded. When in New York go to the American Museum of Natural History and ask to be shown the Peruvian skull, which clearly indicates that life must have been maintained for at least a year, after a fracture involving the lateral region of the skull, the bones uniting to form bridges with large spaces between.

---

Impure Milk. The U. S. Dept. of Agriculuture announces several penalties inflicted for selling milk or cream, either skimmed, adulterated as with annatto, or containing bacillus coli or excessive numbers of ordinary bacteria. These, ot course, apply only to interstate offenses.

---

Abortion Cases. We note with regret, allusions in the newspapers to charges of abortion, one fatal, in our territory. It is contrary to our policy to publish reports of this nature in detail, as, in some cases, a great injustice would be done to innocent victims of circumstance, while, in all, sufficient publicity is given to these unfortunate occurrences in the daily press or in verbal reports to societies. But we feel that a note of warning should be sounded to those tempted to bring discredit upon their profession and ruin upon themselves—not to dwell upon the obvious moral and ethical principles involved.

---

A New Professional Risk. Militant Suffragists in London made an organized raid on physicians' offices October 9, smashing windows along Hanley street, which is a sort of medical row. The logic which led to this demonstration is clear, when it is explained. The home secretary had ordered a resumption of gavage for militants who start hunger strikes; gavage is practiced by physicians; hence the propriety of smashing windows for the profession generally. Whether gastro-enterologists were particularly favored, is not stated. Just imagine the ladies of Buffalo, Rochester, Syracuse, Auburn, Utica, etc., who are demanding the franchise, engaging in a raid of this sort. Most of these ladies are English, barring a few generations of residence in this country; they lead very much the same social and domestic life as the English; they are confronted with approximately the same political problems, so far as their sex is concerned, except that, here, we talk a little louder about liberty and legislate a little more strenuously to prevent a man or a woman from doing as he or she pleases. Two questions suggest themselves: Why do American women who want the ballot continue to behave themselves as respectable, thinking, and, for the most part, feminine members of society, and why do English women present the same argument in a series of foolish and even criminal acts? This question involves an insight into psychology that we lack. The second question is easier to answer: Which set of women will get the vote first?

---

The University of Buffalo has an unusually large registration this year, amounting to over 700 for all departments. The newly established course in arts and sciences has an enrollment of 62, of whom 26 are taking the course as a preparation for medical study. The medical department has the following registration by classes: 90, 44, 54, 47, freshmen to seniors, in order.

---

Extramural and Intramural Typhoid. We have several times called attention to the fact that typhoid is largely a vacational disease, and that this fact explains its autumnal rise in incidence, rather than strictly climatic phenomena; also to the fact that any large city bears the responsibility in its mortality statistics, of sanitary defects outside its limits. The Buffalo Health Department have recently made a study of this phase of the etiology with the following results:

| Month | Cases | Deaths |
|---|---|---|
| June | 11 | 3 |
| July | 15 | 5 |
| August | 29 | 7 |
| September | 53 | 6 |
| Total | 108 | 21 |

| | |
|---|---|
| Not out of city | 35 |
| At summer resorts in Canada | 18 |
| At other parts of Canada | 9 |
| At summer resorts in U. S | 4 |
| At other parts of U. S | 24 |
| Off the lakes | 2 |
| Off the canal | 1 |
| Not stated | 15 |
| Total | 108 |

It cannot be claimed that all of the 58 cases that had been outside the city owed their infection to extramural sources, but, in some instances, an external infection was clearly indicated, notably in the group reported by Dr. C. A. Clements, of three of five persons (one a baby who escaped) infected from a spring in Victoria Park. However, of this group, a non-resident and a resident of Buffalo, were treated out of the city. Allowance must be made for the fact that a person may contract typhoid in Buffalo and be treated, successfully or not, elsewhere, but, on the whole, it is fair to assume that only half of the local typhoid mortality is attributable to local conditions, of which water may or may not be the major source. The fact that 35 persons who had typhoid had not been out of the city, does not necessarily throw the blame on the water supply. Milk is a potent factor in the etiology of typhoid, and we are inclined to suspect some of the bottled waters, imported from springs. Here may be mentioned a very practical point in medical ethics as applied to medical journals. We will not carry the advertisement of any water, or other beverage or food product, without credentials as to its sanitary purity. Whether the price is right, whether there is objection to a mixture of drugs, whether a drug is or is not efficient, whether one should or should not use alcoholic beverages in therapy, are questions on which we think our readers are competent to decide, and we believe that it is not in accordance with general principles of ethics to exclude an advertisement because of a difference of opinion. It should also be stated, as a matter of general ethics, that a great many products that we do not advertise are pure and efficient.

---

The Income Tax. As provisionally settled, a single man is allowed a net income of $3,000 without tax; a married man $4,000. If, however, man and wife have separate incomes, each has an exemption of $3,000. The medical profession should understand that, in general, business expenses are exempt, as is also current expenses of investments and among these expenses, depreciation of property is included. We mention this to anticipate an unfair rating of physicians' earnings as net instead of gross, to point out that office expenses, including a reasonable proportion of rent if the office and residence are combined, as is usually the case, expense for transportation by carriage, buggy, automobile, etc., and expense for drugs, instruments, etc., are strictly business expenses, and, if necessary, to suggest organized defense of the profession against unfair discrimination. Unfortunately, very few physicians will pay the income tax.

---

Bacterial Content of Buffalo Water. The dynamiting of the wreck of the steamer Richardson disturbed the sediment in Lake Erie above the intake to such a degree as to cause a rise in bacterial content to 1120 per c.c., colon bacilli being detected even in samples of 1 c.c. We trust that this is a temporary matter and that the colon bacilli came from the spoiled grain—any grain or agricultural product being liable to contain colon bacilli from manure—and not from sewers. We draw the moral that some form of treatment of the water is desirable and that there should be a reservoir large enough to store water so that the intake need not be used for any ordinary period of agitation of the sediment at the bottom of the lake. And, while the present case is not particularly apropos, we repeat our contention, that, in spite of expense and engineering difficulties, the intake ought to be above the channel of navigation, not below it, as at present.

---

Malta Fever and Goat Milk. The U. S. Dept. of Agriculture have issued a bulletin warning that goat milk should be boiled and claiming that Malta Fever, from this source, has been endemic in Texas and New Mexico for at least twenty-five years. This is contrary to ordinary opinion and deserves careful attention. In advocating the goat as a domestic animal, for various purposes, especially the supply of milk for infants and invalids, and for economic reasons in places where a cow would be too large an animal to harbor, we must not overlook the danger of specific infection nor be so foolish as to steer from the Scylla of tuberculosis to the Charybdis of Malta Fever. On the other hand, it is not at all likely that all goats carry the latter infection. Pending investigation of this point, it is worth while to record the observation that pasteurization at 145 F. for twenty minutes is efficient to destroy the bacterium of Malta Fever.

---

Prohibition. Senator John D. Works of California has introduced a bill prohibiting the sale, manufacture and importation of distilled liquor, containing alcohol, except for mechanic, scientific and medicinal purposes. This bill calls for a constitutional amendment, effective three years after ratification by the legislatures of three-fourths of the States. Speaking with all the prejudice of 120 years of hereditary and personal total abstinence, we beg leave to call attention to the theory that this is a free country and to the objections to reforming anyone by law. We have already reached a point at which there is more interference with personal liberty in this land of freedom, than in any other civilized and enlightened country in the world. Personal liberty obviously implies the liability to do things to which others object, and many of these things are more or less generally considered to be wrong, harmful or unwise. Personal liberty must necessarily stop short of direct damage to the properties and rights of others, but it is wise to allow a wide margin of doubt. A people hemmed in on all sides by restrictive legislation, representing other persons' ideas of right and safety, will retrograde in individual self control and moral sense.

---

State Roads. It is not directly a medical matter but it is one of citizenship and of mortality statistics, that our state roads should be safe and that they should not, after being built by taxation, impose a further tax of direct expense upon those that use them. An ordinary rough country road may jar those who ride over it and increase the wear of vehicles, but a road covered with sharp cornered pieces of flint and wet with oil imposes a very direct expense and danger upon every user of rubber tired vehicles. Bicyclists have some rights, but, for several years, except for the recent limited institution of brick roads, they have been practically excluded from main highways of travel. Tire expense—and it should not be forgotten that a puncture involves actual danger from traumatism, nerve strain, physical strain and exposure—is approximately 50 per cent. greater for those who use parkways and macadamized state roads, as compared with those who use ordinary pavements and country roads. There is no more sense in submitting to the raw condition in which paid officers of the state leave the highways at present than in conceding to a surgeon the right to leave a wound without dressing after an operation.

And we want to add a few words about the Niagara Falls Boulevard. A natural, direct, beautiful route along the river could have been perfected by laying about nine miles and repairing about three miles of brick, following Delaware street to North Tonawanda. Instead of this, about sixteen miles of new pavement was laid, following a route four miles longer, varying from the limit of rural ugliness to mildly picturesque scenery. Starting near the city line with a dangerously curved and rough bit of road, it exhausts the category of irregular curves. By the courtesy of the Automobile Club, danger signs have been placed, but, in a sense, these are themselves a source of danger. Most of the accidents on this road have been due to its narrowness, as it does not provide for three vehicles coming together at once, and to running off at a tangent. The stranger sees the warning of a curve, and sees the curve, and continues at a rate of speed which he considers safe for it. Unfortunately, he does not realize till too late that the curve keeps on winding itself into a coil or else plays snap the whip with the automobile by suddenly turning in the opposite direction. Of course, this constitutes careless driving, but, in a flat country, with no engineering difficulties, no one would expect such a serpentine route.

THE FOURTH ANNUAL TUBERCULOSIS DAY will be observed on Sunday, December 7. Churches, schools, labor unions, fraternal orders, etc., will be asked to prepare special exercises. Over 1000 societies interested in the problem of tuberculosis are behind this movement for popular education. Last year 60,000 churches devoted a service to the subject of tuberculosis, and it is hoped that the number will be increased to 100,000 this year.

---

### Infantile Paralysis

On Wednesday, August 27th, and again on Friday, August 29th, by request, Dr. Roland Meisenbach presented before the Fourth International Congress on School Hygiene some research work pertinent to infantile paralysis. Dr. E. W. Saunders of St. Louis, Dr. Roland Meisenbach of Buffalo, and Dr. W. E. Wisdom of De Queen, Arkansas, have been working on an entirely new theory as to the cause of infantile paralysis. Owing to the fact that the work bears so directly upon the health of the school child, the Congress requested its presentation at this time, although all of Dr. Meisenbach's work is not entirely completed.

Several weeks ago, before a specially called meeting of the St. Louis Medical Society, Dr. Saunders stated that he believed that acute paralysis in children was caused by the injested larvæ of some fly. Dr. Meisenbach stated that it had been known for some time that during epidemics of infantile paralysis there occurred simultaneously disease among animals, chiefly one which had all the characteristics of meningitis; the cause not being known. A few weeks ago there appeared in De Queen, Arkansas, and on the border of Texas, an epidemic of Infantile Paralysis, and at the same time an epidemic of Limber-Neck among the chickens in that locality.

The larvæ taken from the carcasses of these Limber-Neck chickens were collected and fed (not injected) to different animals, namely, monkeys, guinea pigs and chickens, and the results noted. The larvæ were first triturated in glycerine and fed to the animals orally. All of the animals showed signs of an acute paralysis in different degrees, the degree depending upon the dose of the virulent toxic larvæ and the size and resistance of the animal. Guinea pigs became paralyzed in ten to forty-eight hours after receiving five to ten virulent larvæ; monkeys required 106 to 150, and chickens much larger doses. If the dose was large, often death occurred in a short time, due to paralysis of respiration, and the local paralysis was not marked. In the animals that received smaller doses local paralysis manifested itself in twelve to thirty-six, and even forty-eight hours.

The symptoms noted, especially in the monkeys, where they could be accurately observed, were early coryza and lachrymation, with loss of appetite, rise in temperature and rapid respiration. The eyes quickly became slightly clouded, so that it was easy to recognize which animal had received the virulent larvæ. The monkeys showed a profuse nasal discharge and lost their voice; the later symptoms were local motor paralysis, and in some animals subnormal temperature and finally death. In other animals to which the toxic larvæ were given in small doses recovery took place; the latter was especially noted in the chickens. These animals given repeated small doses became somewhat immune.

One of the most convincing animals shown to the Congress by Dr. Meisenbach was the dog. A large Irish setter which inhabited the infected zone was seen to eat the carcasses of the Limber-Neck chickens. He developed a motor paralysis of both hind legs. The paralysis existed thirty days, after which the dog was bled to death for pathological purposes.

This work differs distinctly from anything which has been done in connection with infantile paralysis. The fly theory of others was not directed to the causative factor, as much as to the transmission from the human being to the animals, and from animal to animal by means of the bite. The authors are now working on incubating some of the larvæ. Dr. Meisenbach stated that the research required much work and that the pathology was being done in the most accurate way.

